# Risk factors and efficacy of different intravitreal treatment options for symptomatic focal vitreomacular traction with or without full-thickness macular hole

**DOI:** 10.1007/s10792-025-03591-6

**Published:** 2025-06-12

**Authors:** D. Metzger, A. Assaf, M. M. Maier, S. Groselli, J. Klaas, N. Feucht

**Affiliations:** 1 Eye Medical Care Center, Landshut, Germany; 2 Smile Eyes Airport, Eye Clinic Airport Medical Care Center, Terminalstraße Mitte 18, 85356 Munich, Germany; 3 Deparment of Ophthalmology, Klinikum rechts der Isar, Technical University of Munich (TUM), Munich, Germany; 4 University Eye Clinic, Ludwig Maximilians University, Munich, Germany; 5 Rechts der Isar hospital, Technical University of Munich, Munich, Germany

**Keywords:** Vitreomacular traction, Ocriplasmin, Macular hole, Vitrectomy, VMTS, Pneumatic vitreolysis

## Abstract

**Purpose:**

To report the efficacy and risk profile of intravitreal injections of Ocriplasmin (IVO) versus Perfluoropropane (PVL) in patients with symptomatic focal vitreomacular traction (VMTS) with or without full-thickness macular hole (FTMH < 400 μm).

**Methods:**

Nineteen patients with VMTS received 0.3 ml perfluoropropane, and 68 patients received Ocriplasmin. Primary success criteria included resolution of vitreomacular traction (VMT) and closure of FTMH < 400 μm. Microstructural changes were evaluated using SD OCT for macular hole size, macular edema, subretinal fluid, ellipsoid zone (EZ), and external limiting membrane (ELM).

**Results:**

In the PVL group, 78.92% experienced VMT resolution. None of the FTMH < 400 μm closed with PVL, but all were closed with subsequent pars plana vitrectomy (ppV). New FTMH developed in 7.1% and rhegmatogenous retinal detachment in 5.3%. EZ/ELM changes occurred in 31.6%. In the IVO group, 70.6% achieved VMT resolution. Of 22 patients with FTMH, 45.6% had closure after IVO, with 12 out of 25 needing ppV. New FTMH occurred in 6.5% and retinal detachment in 4.4%. EZ/ELM changes were observed in 16.2%.

**Conclusion:**

Both PVL and IVO showed similar VMT resolution rates. PVL was less effective in closing FTMH and had higher side effects compared to IVO. In the presence of retinal pathologies, PVL is preferable to IVO.

## Introduction

The posterior vitreous detachment (PVD) is the result of a complex and inevitable series of events that occur during the aging process of the eye. It manifests as gel liquefaction and decreased vitreoretinal adhesion [1]. During the aging process of the vitreous gel (synchysis senilis), irreversible restructuring of the collagen-hyaluronate complex occurs, leading to the destruction of the macromolecular network [2]. Simultaneously, biochemical changes at the vitreoretinal interface lead to reduced adhesion strength at the vitreoretinal junction. In abnormal posterior vitreous detachment (APVD), liquefaction is increased, and the vitreoretinal adhesion is not correspondingly decreased [3].

When there is a persistent connection between the vitreous cortex and the retina without altering the anatomical structure of the fovea, it is termed vitreomacular adhesion (VMA). VMA can progress to vitreomacular traction (VMT). VMT causes deformation of the anatomical structure of the fovea due to intense static and dynamic anteroposterior traction forces on the neurosensory retina [4, 5].

The high intensity of axial traction forces on the retina favors the formation of full-thickness macular holes (FTMH). A full-thickness macular hole (FTMH) is defined by an anatomical defect in the fovea, extending through all layers of the neurosensory retina from the internal limiting membrane (ILM) to the retinal pigment epithelium (RPE) [1, 6]. The separation of photoreceptors leads to significant vision loss.

Macular holes are categorized as small (< 250 µm), medium (250–400 µm), and large (> 400 µm) [3, 4, 7]. Additionally, VMT promotes the development of other retinal changes such as pseudocysts, maculoschisis, cystic macular edema (ME), often with increased central retinal thickness, and accumulation of subretinal fluid (SRF). This can impair vision, causing metamorphopsia, micropsia, macropsia, central scotoma, and blurred vision [1, 4].

The greatest treatment success is achieved through surgical release of the adhesion via pars plana vitrectomy (ppV). For VMTS combined with an epiretinal membrane (ERM), VMT > 1500 µm, and an MH > 400 µm, vitrectomy is the therapy of choice, yielding a success rate of 80–100% and MH closure rates of 85–95% [8]. Due to factors such as high costs, the need for an experienced surgeon, anesthesia, inpatient stay, uncomfortable prone positioning for patients, longer follow-up care, and potential complications like risk of bleeding, hypotony, retinal detachment, endophthalmitis (< 1:2000), and early cataract formation et cetera, alternative therapeutic methods are continuously being explored [9].

Two minimally invasive approaches for resolving VMT without ppV are pneumatic and pharmacological vitreolysis. Pneumatic vitreolysis (PVL) is a promising therapeutic option due to its accessibility and low cost. It involves the intravitreal injection of gas, which has been widely used in the treatment of retinal detachment and smaller peripheral retinal tears in the pneumatic retinopexy procedure [10]. For VMT Chan et al. reported successful treatment results with intravitreal gas injection of perfluoropropane (C3F8). A single gas injection resolved 95% of VMTs and closed up to 50% of stage 2 MHs in their study [11]. Positive results were confirmed by further studies, showing PVL led to VMT resolution rates of 50 to 100% [12], low postoperative complications [13], and approximately 50% closure rates of macular holes [14]. Unlike pharmacological vitreolysis, traction on the fovea is solely resolved by the expansive properties of the highly concentrated gas bubble.

However, the previously considered low side effect profile of PVL is increasingly questioned. In a study by Baumann et al. [15] in 2022, new macular holes occurred in 12.1% of cases, 71.4% of MHs remained unclosed, and 8.5% of patients developed retinal detachment. Despite a high VMT resolution rate, patients with additional MH should be informed about the high likelihood of subsequent ppV. Chan et al. in 2021 [16] prematurely terminated their prospective study due to concerns about the increased risk of retinal detachment from PVL. Despite growing skepticism about the use of PVL and a MH closure rate of 29%, PVL remains a therapeutic option for VMTs, according to Chan et al.

Given the generally small incidence patient population and lack of higher evidence levels, PVL is rarely practiced in clinical routine [14].

**Fig. 1:**
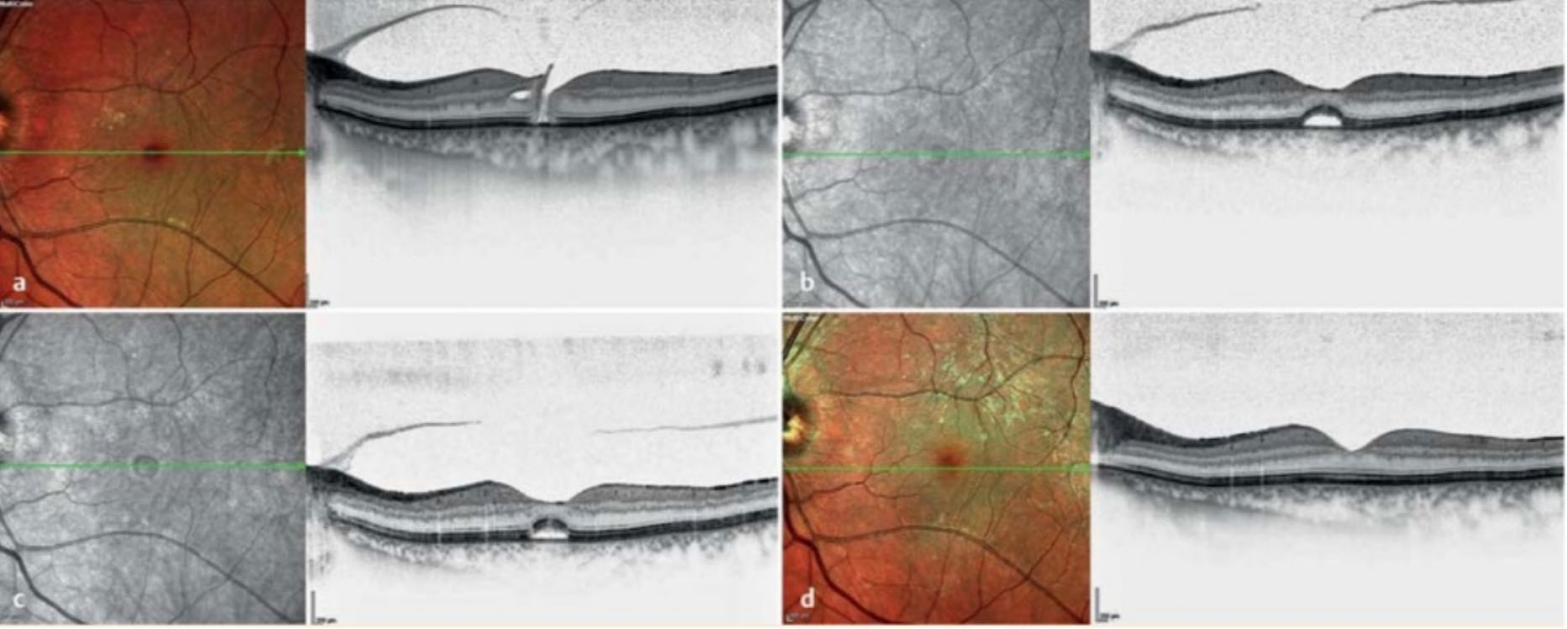
Follow up of a vitreomacular traction case (VMT) with a small macular hole after an intravitreal injection of Ocriplasmin (IVOM). **a** Initial findings in the left eye: VMT with a small macular hole < 250 µm, visual acuity 0.5, and metamorphopsia. **b** 1 month after intravitreal Ocriplasmin injection (IVOM): macular hole closed, subfoveal fluid present, visual acuity 0.8 (with correction). **c** 4 months after IVOM: macular hole closed, subfoveal fluid receding, visual acuity 0.8 (with correction). **d** 10 months after IVOM: macular hole closed, subfoveal fluid absorbed, visual acuity 0.9, cellophane reflex on the multicolor image (left)

Since 2013, the drug Ocriplasmin (Jetrea^®^, ThromboGenetics N.V. ©) has been officially approved by the European Medical Agency for pharmacological vitreolysis of symptomatic VMT with small full-thickness macular holes < 400 μm [17]. The recombinant serine protease induces PVD through proteolytic action on fibronectin and laminin, both key components of the vitreomacular interface [18]. The effectiveness of a single injection of 0.125 mg/0.1 ml in detaching VMA is reported between 24 and 66.6% [19, 20]. The closure rate of small MHs varies between 17 and 80% depending on the patient population [21, 22] (Fig. 1). Despite sufficient randomized clinical data and a defined suitable patient profile, limitations in patient selection such as MH < 400 µm, VMT < 1500 µm, absence of ERM, and other retinal pathologies like diabetic retinopathy, as well as high costs and the drug’s side effect profile, limit the use of Ocriplasmin as a primary therapeutic option in clinical practice [7, 23].

The primary objective of this study is to compare two promising therapeutic options regarding their risk profiles and treatment success for VMTs with or without FTMH < 400 µm. Patient profile preselection is crucial for treatment success. A better understanding of the success parameters for both PVL and IVO should aid clinical decision-making. Choosing the appropriate method can enhance treatment success and minimize side effects. Ultimately, ppV following Ocriplasmin or gas has not proven disadvantageous, but unnecessary delays in surgical intervention and the associated risk of significant vision impairment should be avoided [23].

## Methods

In this study, retrospective real-life data analyses were conducted on patients treated for symptomatic VMT with or without a full-thickness macular hole (FTMH) < 400 μm. The treatments consisted of either an intravitreal injection of 0.3 ml perfluoropropane gas C3F8 (19 patients) or an intravitreal injection of 0.125 mg/0.1 ml Ocriplasmin (68 patients) at the ophthalmology department of the University Hospital of the Technical University of Munich, Rechts der Isar, between January 2013 and May 2020.

Inclusion criteria for both treatments were a VMT ≤ 1500 μm with or without FTMH ≤ 400 μm, provided the VMT was untreated and there were no other retinal conditions such as an epiretinal membrane (ERM), diabetic retinopathy, or macular degeneration. The clinical diagnosis was confirmed by funduscopy and SD-OCT scans (Spectralis HRA + OCT, Heidelberg Engineering, Heidelberg, Germany). To rule out an MH, an additional radial OCT of the fovea was performed.

Microstructural morphological changes were recorded in the horizontal 19-line OCT scan or the radial scan centered on the fovea on the day of the injection, including VMT diameter, EZ and ELM changes, presence of ME, and SRF. If an MH was present, the minimal linear diameter at the narrowest point was measured. Vitreomacular adhesion and central retinal thickness were also measured.

Complications and functional visual acuity (VA) changes were recorded, and general variables such as age, sex, lens status, and visual acuity were considered. The resolution of VMT and closure of MH were set as primary success markers, and changes were evaluated after one week, one month, three months, six months, one year, and at the last follow-up (LFU).

## Results

The PVL group had 19 patients, including 10 women, with 31% being pseudophakic. In the IVO group, with 68 patients, 73% (50) were women and 48.5% were pseudophakic. There were no significant differences between the groups in terms of sex, lens status, and age, as determined by t-tests and chi-square tests. the same surgeon operated on all patients (Table 1).

**Table 1:** Demographic data: age and gender distribution of the study participants

	IVO		PVL	
	VMT resolved	VMT not resolved	VMT resolved	VMT not resolved
Age (years)				
Median	71	77	71	75
1/4 quartile	63	69.75	67	70.78
3/4 quartile	76	82.25	77	79.5
IQR	13	12.5	10	8.75
Gender				
Male	9	9	7	2
Female	39	11	8	2

**Fig. 2:**
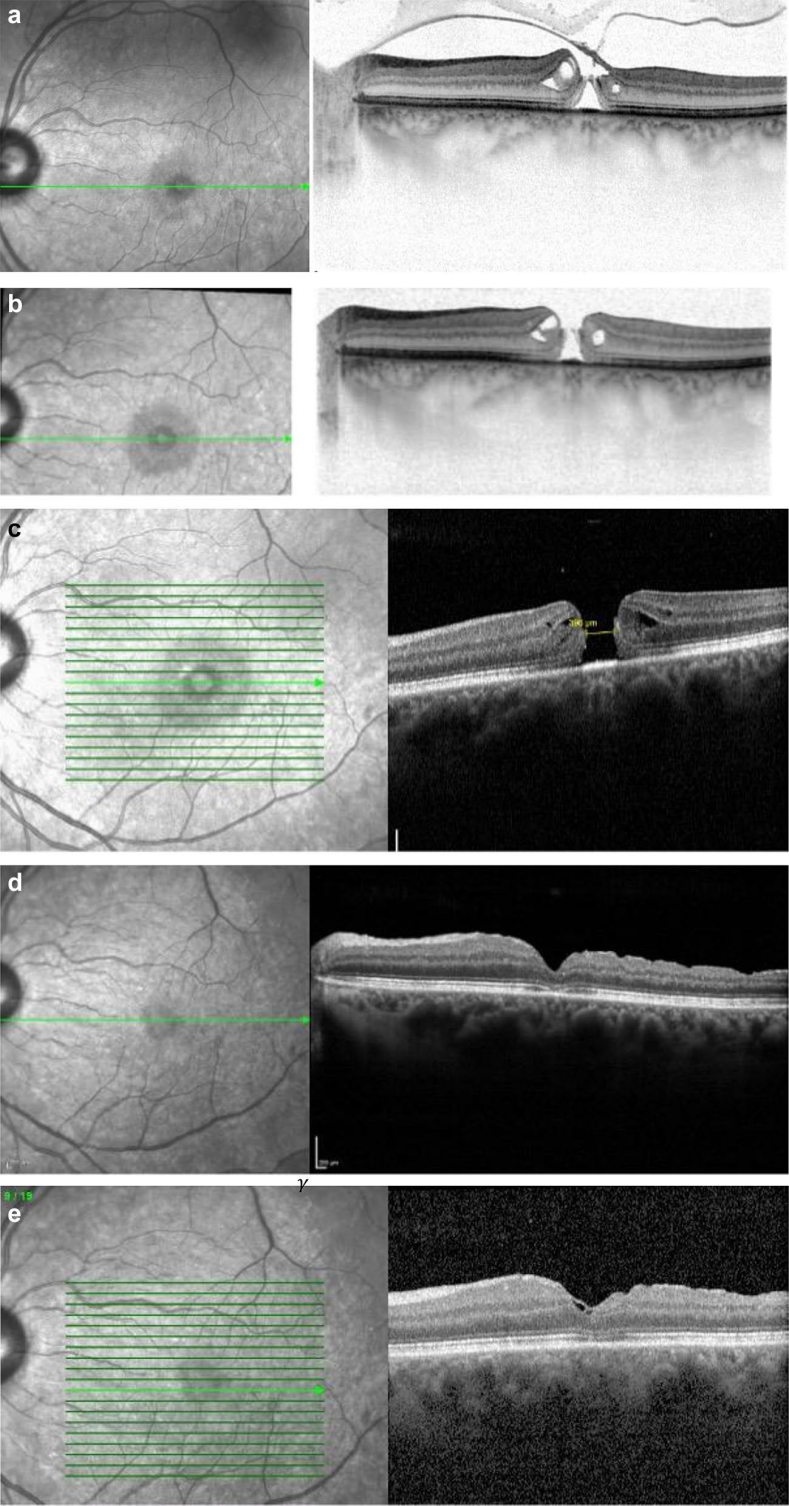
Progression of a VMT with a small macular hole following pneumatic vitreolysis (PVL) and subsequent pars plana vitrectomy (PPV). **a** Baseline condition of the left eye, horizontal OCT image: incomplete posterior vitreous detachment, focal vitreomacular traction (VMT) with a macular hole (MH) and peripapillary adhesion, intraretinal cysts, visual acuity (VA) 0.4. **b** Two weeks after pneumatic vitreolysis (PVL) with intravitreal injection of 0.3 ml C3F8: VMT resolved, intraretinal fluid present, macular hole (MH) increased in size (241 µm), visual acuity (VA) 0.3 with pinhole. **c** 19 OCT scan, 2 months after PVL: Decrease in intraretinal fluid, macular hole (MH) size increased (396 µm), visual acuity (VA) 0.2. **d** 2 months after pars plana vitrectomy (PPV), internal limiting membrane (ILM) peeling, and gas injection: macular hole (MH) closed, intraretinal fluid absorbed, epiretinal membrane (ERM), subfoveal interdigitation zone not continuous; visual acuity (VA) 0.4 pp. **e** 6 months after pars plana vitrectomy (PPV), internal limiting membrane (ILM) peeling, and gas injection: macular hole (MH) closed, outer segments in restitution, visual acuity (VA) 0.6 p

**Fig. 3:**
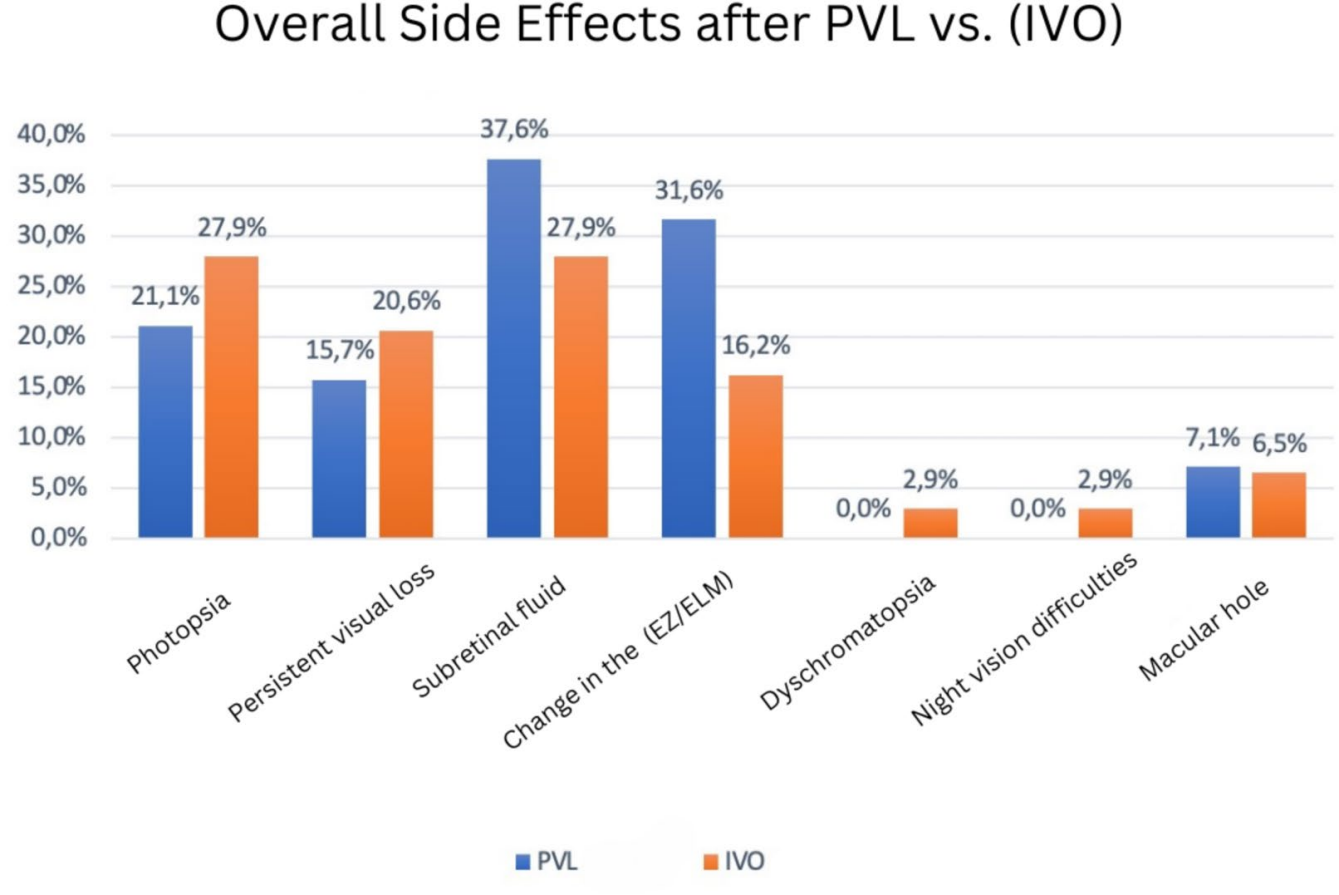
Comparison of therapeutic outcomes after intravitreal injection of Ocriplasmin (IVO) and pneumatic vitreolysis (PVL) with 0.3 ml C3F8

In 15 out of 19 patients (78.9%) treated with PVL, a VMT resolution was achieved (Fig. 3). Four of the 15 VMTs resolved by the first day, and an additional seven resolved within two weeks (13/15). Five of the 14 patients (35.71%) with VMT without FTMH subsequently required ppV. In 7 out of 15 patients (46.7%) with resolved VMT, a reduction in retinal thickness was observed. Preoperatively, an FTMH < 400 μm was detected in 5 out of 19 patients (26.3%). All 6 patients with an FTMH required subsequent ppV as none of the holes closed with the gas injection. After three months, 4 out of 6 patients underwent vitrectomy, and by six months, all macular holes were successfully closed by ppV (Fig. 2a–e; Table 2).

**Table 2:** Treatment outcomes: observed treatment responses, including resolution of vitreomacular traction, macular hole closure, and the need for subsequent pars plana vitrectomy (ppV)

Therapy success IVO vs. PVL	%	%
	IVO	PVL
VMT-resolution	71	79
MH- closure	46	0
subsequent ppV	35	58

One patient with resolved VMA after PVL developed a new FTMH (5.3%). One patient developed a retinal detachment (5.3%).

Overall, due to VMT, persistent MH, and retinal detachment, 11 out of 19 patients (57.9%) underwent ppV. Visual acuity (VA) reduction was reported in 11 out of 19 patients (57.9%), 8 transient (42.1%), and 3 persistent (15.8%). Persistent VA reductions occurred after retinal detachment, epithelial haze, and a central blind spot due to an FTMH. Transient VA reductions occurred in 6 out of 8 (75%) VMT resolved cases. Metamorphopsias were reported by 16 patients, 8 after the injection, with 2 having persistent VMT.

Pseudophakia did not show any advantage in therapeutic success in this patient group. Six patients were pseudophakic at the time of injection. VMT resolution occurred in 4 out of 6 pseudophakic patients (66.7%) and in 11 out of 13 phakic patients (84.6%).

Preoperatively, macular edema was present in 16 patients (84.2%), which reduced to 50% after one month and 30% after six months, all post-ppV. Subretinal fluid (SRF) was present in 10 out of 19 patients (52.6%) preoperatively, with 30% persisting, all post-ppV. SRF developed in 6 patients (31.6%) after gas injection. At the six-month follow-up, SRF was observed in 18.8% of the patients.

**Fig. 4:**
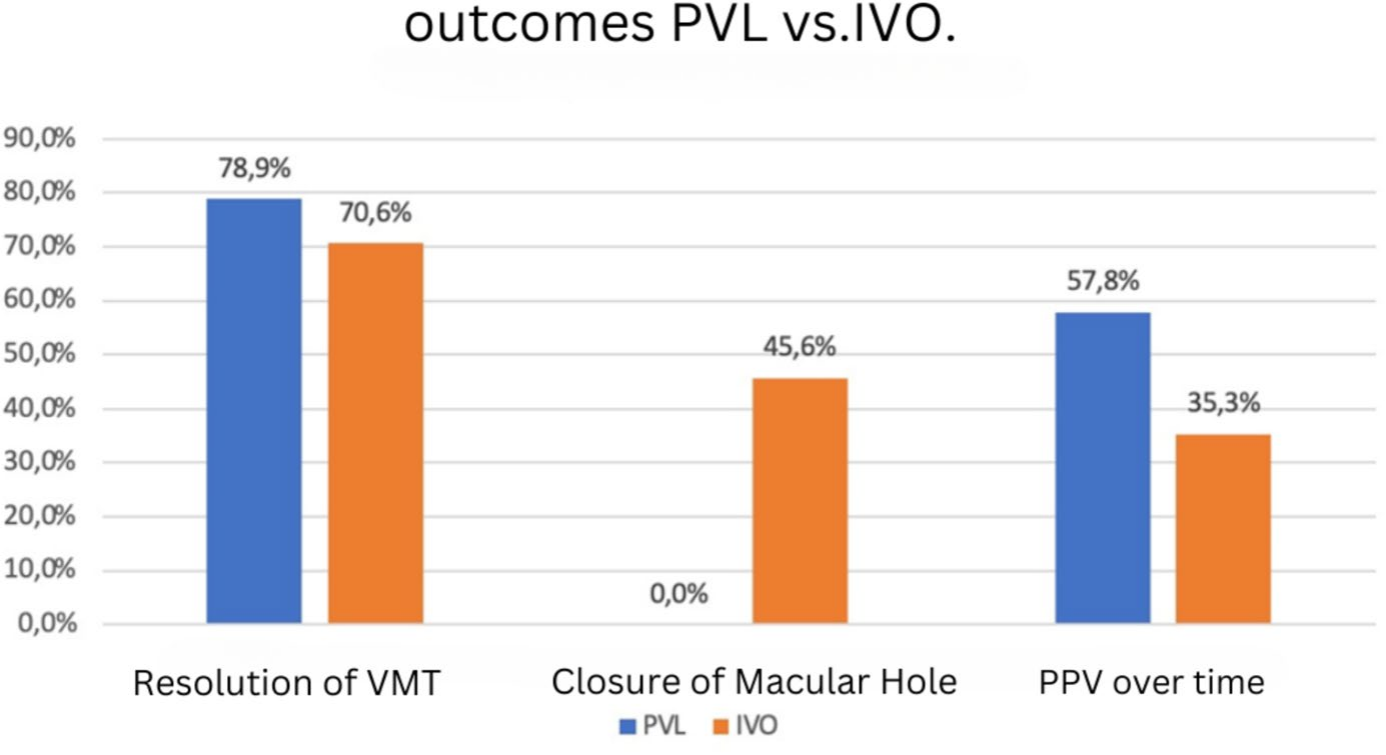
Comparison of side effects in patients after intravitreal injection of Ocriplasmin (IVO total) and pneumatic vitreolysis with 0.3 ml C3F8 (PVL total)

Pre-injection ELM changes were present in 8 out of 19 patients (42.1%), and EZ changes in 11 out of 19 patients (57.9%). Two patients developed EZ/ELM changes with VMT resolution (10.52%), with one patient having an outer segment defect persisting after one year. With persistent VMT, 4 patients (100%) developed outer segment defects (4EZ/2ELM). Post-ppV, EZ changes persisted in 2 patients. Unlike the Ocriplasmin patient group, no dyschromatopsias or night vision difficulties were reported. Three out of 19 patients considered the gas bubble a nuisance. Two patients experienced an intraocular pressure increase above 20 mmHg, one of whom had a retinal detachment. The side effects following PVL are listed in Fig. 4.

For 48 out of 68 patients treated with an intravitreal injection of Ocriplasmin, VMT resolution occurred. Eight out of 46 patients (17.39%) with VMT without FTMH subsequently required ppV. A reduction in retinal thickness was observed in 34 out of 48 patients (70.8%) with resolved VMT. Preoperatively, an FTMH < 400 μm was detected in 22 out of 68 patients (32.3%). The macular hole closure rate was 45.6% (10 out of 22 patients) (Fig. 3).

Three patients with resolved VMT developed a new FTMH (6.5%). Overall, 12 out of 25 patients with FTMH underwent surgical treatment via pars plana vitrectomy (ppV) (48%). After one month, 5 patients underwent vitrectomy, and after six months, all vitrectomized macular holes were successfully closed. Three patients developed a retinal detachment (4.41%).

In total, 24 out of 68 patients (35.3%) underwent ppV. Visual acuity reductions were reported in 34 out of 68 patients (50%), with 14 persistent (20.6%) and 20 transient (29.4%). Among the 14 patients with persistent visual acuity reductions, 6 had unresolved VMT. Seventeen out of 20 transient visual acuity reductions occurred after VMT resolution. Metamorphopsias were reported by 44 patients, 6 after the injection, with 2 having persistent VMT.

Among the 48 successfully treated patients, 20 were pseudophakic (41.70%) and 28 phakic (68.30%). Preoperatively, macular edema was present in 59 patients (86.8%), reducing to 66.6% after one month and 25% after six months. Subretinal fluid was present preoperatively in 38 out of 68 patients (55.9%), with 7 persisting at the six-month follow-up (10.29%). SRF was observed in 19 patients post-injection (27.9%). Overall, SRF was present in 19% of patients at the six-month follow-up.

Pre-injection ELM/EZ changes were present in 52 out of 68 patients (76.47%). Post-injection, 11 patients developed changes in these zones (16.2%), with 3 having persistent VMT. Dyschromatopsias and night vision difficulties were reported by 2 patients each (2.9%). The side effects following IVO are listed in Fig. 4, Table 3.

**Table 3:** Adverse events: reported complications and adverse effects observed in the study cohort, including visual disturbances and structural retinal changes

Functional side effects IVO vs. PVL	%	%
	IVO	PVL
Photopsy	28	21
Persistent VA reduction	21	16
SRF	28	38
EZ/ELM disruptions	16	32
Dyschromatopsy	3	0
Night vision reduction	3	0
MH-formation	7	7
Retinal detachment	4	5

## Discussion

### VMT resolution rates

The VMT resolution rates of 70.6% for IVO and 78.9% for PVL confirm that patients with a VMT less than 1500 µm benefit from both minimally invasive methods. The intravitreal injection of gas proved to be the more effective method for resolving vitreomacular traction with a success rate of 78.9%. Further studies report even higher VMT resolution rates following PVL [12, 24, 25]. The results from the comprehensive meta-analysis by Yu et al. showed that PVL was most effective for VMT resolution at 85%, followed by Ocriplasmin at 43.9%, which is below our results for Ocriplasmin [25].

### FTMH

For cases with an FTMH less than 400 µm, PVL was found unsuitable in this analysis group. Previously, Özdemir et al. reported an MH closure rate of 0% after PVL [12]. Yu et al. also reported a lack of MH closure following PVL [25]. Higher closure rates of FTMH less than 400 µm with PVL were shown by Baumann et al. (28.6%) and Chan et al. (29%) [15, 16].

Our analysis showed significantly higher treatment success for MH closure with IVO at 45.6%. Yu et al. [25] reported an MH closure rate of 33% following IVO treatment, which is below our results and about half the rate reported by Groselli et al. at 60% [26].

### Risks (MH + retinal detachment)

In 7.1% of cases following PVL, new MH developed. A progression in the size of MH was noted, similar to Baumann et al.’s findings [15], where the incidence of new MH was 12.1%, higher than in our analysis [15]. Yu et al. (2016) also reported new MH formation in 4.1% of cases after PVL [25].

After the application of Ocriplasmin, 6.5% of patients developed new MH, which falls within the previously reported range of 3.8–8% [25, 26].

The risk of retinal tears is also significant with induced vitreolysis. Following pneumatically induced traction detachment, 5.3% of cases resulted in retinal detachment. Further studies reported the same side effect after PVL (8.5–12%) [15, 16]. In contrast to studies that found no association between PVL and retinal tears or detachment [22, 26], the prospective study by Chan et al. [16] on PVL in patients with VMT and MH had to be terminated early due to retinal aberrations occurring more frequently than expected. The combined results of two studies (VMT with and without MH) showed a rhegmatogenous retinal detachment rate of 12% [16]. This is significantly higher than the previously mentioned incidence rates [12, 15, 24, 25, 27]. Only one-third of the MH cases were closed in Chan et al.’s study, with a 94% VMT resolution rate achieved 24 weeks after the gas injection. According to Chan et al., the high treatment success justifies PVL in clinical practice for patients with VMT without MH [16].

In our PVL data analysis, 100% of patients with FTMH required surgical closure of the MH despite resolved VMT. For VMT without MH, only 35.7% required subsequent ppV.

Following Ocriplasmin injection, 48% (12 out of 25) of patients with MH needed subsequent ppV, including 8 patients despite resolved VMT. Only 21.7% (10 out of 46) of patients with VMT without MH required ppV, including 3 due to retinal detachment. This suggests that ppV should be preferred in the case of MH to preemptively counteract vision deterioration.

### EZ/ELM changes

Changes in the outer segments of the retina in the OCT, specifically the EZ/ELM, are frequently associated with visual acuity reduction [28]. In our study, 50% of patients experienced visual acuity reduction following intravitreal Ocriplasmin injection, with slightly more than half of these reductions being transient. In contrast, 16.2% of all IVO-treated patients showed EZ/ELM changes, likely due to the proteolytic properties of Ocriplasmin.

After gas injection, 31.6% of patients showed changes in the EZ/ELM segments, compared to 16% following IVO. A possible cause might be the uneven distribution of the gas bubble on the macula, particularly in the initial weeks post-PVL. The described mechanical effect could result from both the gas bubble and the Ocriplasmin injection. Postoperative inflammation after ppV can also have negative impacts on the EZ/ELM layers.

The restoration of the outer segments of the retina correlates with a positive effect on visual acuity, as noted in the study by Sun et al. [28]. In this study, the ELM was restored in most cases after one month, while the EZ took the longest to recover [29]. This is confirmed by our results, where EZ changes occurred more frequently and persisted longer than ELM changes. A potentially negative effect of Ocriplasmin on the photoreceptor layer of the retina is supported by the fact that dyschromatopsia and night vision impairment were observed only after pharmacological vitreolysis, albeit each at 2.9%.

### Other side effects

Literature associates Ocriplasmin with not only visual acuity reduction but also field constriction with electroretinogram signal changes, retinal vessel narrowing, and pupil anomalies [21, 26, 30].

After intravitreal gas injection, the increased risk of early cataract development is a significant consequence [31]. Particularly in younger, phakic patients, the anatomical situation should be considered in therapy decisions. In our study, no early cataract formation was observed during the 12-month observation period. Other studies support these findings [10, 12, 14, 24, 32]. The "Face-Down" head position in the initial days following injection enhances therapeutic success and minimizes contact between the gas bubble and the lens, aiding in cataract prevention. Additionally, the injected gas volume is significantly less than with complete vitreous replacement in standard vitrectomy.

### Positive predictors

A high treatment success with Ocriplasmin can be achieved through targeted patient selection. The impact of preselection is highlighted by varying results in previous studies. The VMT resolution rate with IVO in the first Phase III study in 2012 was 26.5% [33]. By excluding cases with ERM, broad VMT (> 1500 µm), and other retinal pathologies such as diabetic retinopathy, higher success rates were achieved in other studies [23, 26].

Our study’s patient cohort was selected based on the same inclusion criteria for PVL treatment as for Ocriplasmin. Patients with ERM, VMT > 1500 µm, or other retinal pathologies were excluded. The presence of ERM can promote stronger adhesion of the macula to the vitreous due to fibrocellular proliferation [34]. The presence of these pathologies is already an exclusion criterion for Ocriplasmin treatment. In cases with ERM, Ocriplasmin achieved 8.7% VMT resolution compared to 37.4% in patients without ERM [33].

Given the limitations in the patient cohort and drug-associated side effects, Ocriplasmin is used less frequently despite its high therapeutic success.

The study by Stainle et al. [24] included patients with diabetic retinopathy (DR) and ERM for PVL treatment. The treatment success rate for DR patients was 90%, and for eyes with ERM, it was 83%, significantly higher than Ocriplasmin combined with these pathologies. Additionally, patients previously unsuccessfully treated with IVO were given intravitreal gas injections, with 83% subsequently resolved by PVL.

In DM patients with DR, fibrous vascular ingrowths in the posterior vitreous can favor small retinal tears. The high PVL success rate in DR patients in the mentioned study is promising, potentially minimizing complications and reducing the risk of retinal detachment in DR patients.

Positive predictors for a favorable outcome with PVL include pseudophakia and younger age.

Baumann et al. found significant differences in VMT resolution between pseudophakic eyes (100%) and phakic eyes (67.6%) with PVL [15]. A similar study by Fouad et al. observed that pseudophakic eyes achieved a 90% VMT resolution rate with SF6 gas, with an overall resolution rate of 80% [32]. The positive effect of pseudophakia on treatment success can be explained by the anterior shift of the vitreous body post-lens surgery [35]. Our results for PVL showed no advantage for pseudophakia, with 50% of resolved VMT cases being pseudophakic.

For Ocriplasmin treatment, there is high evidence that phakic lens status is a positive predictive factor for therapeutic success [17, 19, 33, 34, 36]. This is confirmed in our Ocriplasmin data analysis, with an 80% VMT resolution rate in phakic patients versus 60% in pseudophakic patients.

Other studies identified younger age as a statistically significant predictor for Ocriplasmin success [35, 37]. In our analysis, the median age for therapeutic success with IVO and PVL was 71 years. When therapy failed post-Ocriplasmin injection, the median age was 77 years, and 75 years post-PVL. There is a correlation.

### Limitation

The differences in group sizes are due to our predefined study period and recruiting procedure, during which all patients who underwent surgery were included.

## Conclusion

Precise patient selection plays a significant role in therapeutic success. For broad VMT with a diameter > 1500 µm and MH > 400 µm, as well as in the presence of an epiretinal membrane (ERM), pars plana vitrectomy (ppV) remains indispensable and the treatment of choice. For isolated vitreomacular traction < 1500 µm, after thorough patient education about the risks of complications, treatment with pneumatic or pharmacological vitreolysis can be considered. In the case of additional retinal pathologies, pneumatic vitreolysis is preferable to pharmacological vitreolysis. For VMTS with an additional small macular hole, treatment with Ocriplasmin is viable, provided the patient is thoroughly informed about the risks and alternative treatment options. However, PVL is not recommended.

## Summary

### Previously known

- Pharmacological vitreolysis is suitable for treating vitreomacular traction syndrome (VMTS) with or without a full-thickness macular hole (FTMH) < 400 µm.
- Retinal pathologies such as ERM, diabetic maculopathy, or large retinal holes are exclusion criteria for pharmacological vitreolysis.
- Pneumatic vitreolysis is another minimally invasive method with high success rates for treating VMTS.

### New findings

- PVL is not recommended for treating VMTS with small FTMH < 400 µm.
- The side effect profile of PVL is higher than in comparable studies, including the development of new macular holes, progression in macular hole size, persistent EZ/ELM changes, and rhegmatogenous retinal detachments.
- PVL is not recommended for treating VMTS with small FTMH < 400 µm.
- The side effect profile of PVL is higher than in comparable studies, including the development of new macular holes, progression in macular hole size, persistent EZ/ELM changes, and rhegmatogenous retinal detachments.

## Data Availability

The data supporting the findings of this study are available upon reasonable request. The study was conducted as part of a doctoral thesis at the Technical University of Munich, University Hospital rechts der Isar. Due to institutional policies and patient confidentiality agreements, access to the data may be restricted. Researchers interested in accessing the data should contact the corresponding author.
